# Hemp Seeds of the Polish ‘Bialobrzeskie’ and ‘Henola’ Varieties (*Cannabis sativa* L. var. *sativa*) as Prospective Plant Sources for Food Production

**DOI:** 10.3390/molecules27041448

**Published:** 2022-02-21

**Authors:** Mirosława Teleszko, Adam Zając, Tomasz Rusak

**Affiliations:** 1 Department of Food Technology and Nutrition, Wroclaw University of Economics and Business, Komandorska 118/120 Street, 53-345 Wroclaw, Poland; 2 Department of Bioorganic Chemistry, Wroclaw University of Economics and Business, Komandorska 118/120 Street, 53-345 Wroclaw, Poland; adam.zajac@ue.wroc.pl; 3 BIOTRECO Sp. z o.o., Łąkowa 2C Street, 55-040 Bielany Wrocławskie, Poland; tomasz.rusak@biotreco.pl

**Keywords:** hemp seeds, nutrition value, fatty acids, amino acids, plant-based food

## Abstract

This publication characterizes the nutritional value of the Polish hemp seeds of the ‘Bialobrzeskie’ and ‘Henola’ varieties, including the profile/content of fatty acids and amino acids. Hemp seeds were found to be rich in protein, fat, and dietary fiber. Polyunsaturated fatty acids (PUFA) dominated the unsaturated fatty acids (UFA) profile. Their average share within the total fatty acids (FA) was as high as 75%. Linoleic acid belonging to this group accounted for 55% of the total FA. Lipid profile indices (Σ *n* − 6/Σ *n* − 3, Σ PUFA/Σ SFA, the thrombogenicity index, the atherogenicity index and the hypocholesterolemic/hypercholesterolemic ratio) proved the high nutritional value of hemp oil. Considering the tyrosine + phenylalanine and histidine contents, hemp protein exhibited a great degree of similarity to egg protein, which is known and valued for its high biological value.

## 1. Introduction

According to the European Industrial Hemp Association (EIHA) report of 2018, industrial hemp (*Cannabis sativa*) production on the Old Continent covers an area of 50,081 ha and the yearly average has increased by over 600% compared to the production recorded in 1993 [1]. The usable parts of hemp include leaves and flowers that are used, for example, for the manufacture of food supplements (58%), essential oils (20%) and tea (6%), as well as for medical applications (1%) and other uses, e.g., in the cosmetic industry (15%). The primary seed processing lines include the production of hemp flour, animal feed, fiber preparations, husked seeds, and food, mainly oil.

According to analysts from New frontier Data [2], Poland has significant potential to become one of the key producers of hemp in Europe. There are many arguments to support this. The acreage of *Cannabis sativa* in Poland recorded an 80% increase in 2018–2020 and now totals around 3000 ha. The sales value of industrial hemp farming amounted to USD 9.6 million, providing a raw material base for the textile, food, cosmetic and construction industries. Essential market players, such as Canopy Growth, Aurora and Australian GC Pharma, started recognizing the promising opportunities in the Polish hemp industry [2]. At present, the Polish National List of Agricultural Plant Varieties includes 11 varieties of industrial hemp, i.e., the ‘Beniko’, ‘Bialobrzeskie’, ‘Glyana’, ‘Henola’, ‘Mietko’, ‘Rajan’, ‘Sofia’, ‘Tigra’, ‘Wielkopolskie’ and ‘Wojko’ monoecious varieties, as well as the ‘Matrix’ dioecious variety. They all exhibit tetrahydrocannabinol (THC) content in the dry matter of the inflorescence mass not exceeding 0.2% [3].

In recent years, the global economic expansion of hemp has taken several directions. The considerable interest in the medicinal applications of this plant is based on the centuries-old experience of using *Cannabis sativa* in the folk medicine of China, Assyria, Egypt, and ancient Greece and Rome [4,5]. Already 5000 years ago, the first Chinese pharmacopeia by the emperor Chen Nung mentioned prescribing cannabis as a drug for fatigue, malaria and rheumatism [6]. Hemp’s phytocannabinoids, including Δ-9-tetrahydrocannabinol (Δ-9 THC) and cannabidiol (CBD), and their therapeutic properties, are the subjects of research in many scientific centers worldwide. Their thematic scope is diverse and includes, among others, the assessment of the potential use of these compounds in treating neurological diseases (including epilepsy) [7,8], mental disorders (including anxiety, clinical depression and sleep disorders) [9], pain [10,11], cancer [12], rheumatoid arthritis [13] or Crohn’s disease [14].

Food production is also an essential and highly prospective direction for the use of hemp, particularly with the dynamically developing vegan food industry in mind. The desire to limit the production and consumption of meat worldwide is undeniable. According to the European Commission’s forecasts [15], meat consumption in the Community will decrease from 68.7 kg per capita in 2020 to 67.6 kg per capita in 2030. This decrease will mainly result from a change in consumer preferences and a reduction in the consumption of red meat. As Aschemann-Witzel et al. [16] noted, the growing global demand for protein, its insufficient consumption in certain population groups, as well as the numerous advantages of plant protein (health aspects, sustainable development, etc.) have provided an impulse for the development of modern protein products of plant origin and contributed to the growing popularity of plant products and diets. In this context, hemp seeds appear to provide a lot of application opportunities. This raw material is rich in easily digestible protein (20–25%) with a favorable amino-acid profile. It also contains 25–35% lipids with a unique and perfectly balanced fatty acid composition. The share of carbohydrates is high at 20–30%, with a significant proportion of this fraction being dietary fiber (primarily insoluble) [17]. Hemp seeds are also a valuable source of many mineral components (e.g., P, K, Mg, Ca, Na and Fe) [18].

Given the considerations presented above, the purpose of this work was to determine the nutritional value of hemp seeds of two Polish varieties, ‘Henola’ and ‘Bialobrzeskie’, and to determine their possible use for the production of valuable plant products.

## 2. Results and Discussion

### 2.1. Nutritional and Energy Value of Hemp Seeds

Seeds of the tested hemp varieties exhibited similar nutritional quality parameters (Table 1; *p* < 0.05). They contained 32.52% fat (mean value), 23.47% protein, 2.05% total sugars and 4.66% total ash. The digestible carbohydrates content of the ‘Henola’ variety was greater than estimated for the ‘Bialobrzeskie’ variety (4.48%). However, in ‘Bialobrzeskie’ seeds, a higher content of dietary fiber was noted (28.88%). Hemp seeds thus have a valuable chemical composition. The analysis of their main features already shows a broad spectrum of potential raw material applications in food production.

Our research confirmed that fat is a crucial component of hemp seed. As read in the literature, its share in seeds is subject to several factors, including plant genotype [19], climate and agronomic conditions [20]. Vonapartis et al. [21] found that the fat content in seeds of 10 hemp varieties grown in Canada ranged from 269 (‘Delores’) to 306 g/kg of dry matter (DM; ‘Finola’). Lan et al. found higher oil levels in the analyzed hemp seeds [22]. In the ten varieties from North Dakota crops, USA, tested by Lan et al., fat accounted for between 32.75 (‘X-59’) and 35.88% of dry matter (‘CFX-1’). Thus, similar results were obtained in our research on Polish ’Henola’ and ‘Bialobrzeskie’ varieties (Table 1). From the study published by Mihoc et al. [23], it follows that varieties grown in Romania were much less rich in fat. Depending on the harvesting season (2010 and 2011), the oil content of the seeds ranged from 20.82 (‘Diana’) to 29.27% (‘Armanca’). However, these authors pointed out that the monthly average temperatures in the flowering period combined with low rainfall levels could contribute to the incomplete ripening of the seeds and decreases in their oil content. In turn, from the experiment of Alonso-Esteban et al. [24], it was found that of the eight hemp varieties examined, ‘Fedora 17’ was the richest in oil (32.24%). In the ‘Bialobrzeskie’ variety, its content did not exceed 29%. These observations thus confirm that, in addition to variety, the geographical crop location and the resulting soil and climate conditions also significantly impact the fat content in *Cannabis sativa* seeds.

From the nutritional quality and potential health advantages point of view, hemp oil is considered to have a beneficial effect on human metabolism, which is linked not only to the high content of polyunsaturated fatty acids (PUFA) from the *n* − 3 and *n* − 6 families, but also to their proportions [25]. We discuss this topic in detail in the section entitled “Fatty Acids Profile and Lipid Indices”.

It follows from our research that hemp seeds of the ‘Henola’ and ‘Bialobrzeskie’ varieties are a valuable source of protein, with their contents exceeding 23% in both varieties. These values are similar to those determined for other industrial hemp varieties by House et al. [26], Vonapartis et al. [21] and Siano et al. [27]. As Wang and Xong [28] noted, due to their high nutritional value, hemp protein is increasingly gaining interest in research centers worldwide, reflected in a dynamically growing number of publications on it. Hemp has good application potential for producing the so-called protein flour, isolates and protein concentrates [29], and drinks such as plant “milk” [30].

*Cannabis* seed mainly consists of storage proteins, including albumins (25–37%) and edistin/globulin (67–75%). They do not contain protease inhibitors, which results in improved digestive properties of the protein. The content of essential amino acids in hemp seed proteins is higher than in those of soya and is sufficient for people of ten years and older. The amino acid profile of the proteins found in seeds of the ‘Henola’ and ‘Bialobrzeskie’ varieties has been described in detail in the “Amino Acids Profile and Content” section.

High fiber content also needs to be mentioned when assessing the nutritional quality of hemp seeds. In the studied material, the fiber content was 28.88 and 27.42% (Table 1; *p* < 0.05). According to other authors, these values range from 27.6% to as much as 33.8% [18,31]. Among other things, fiber-rich hemp can be used to create new bakery formulas. In their research, Korus et al. [32] successfully used hemp flour as an additive to starch to bake gluten-free bread, which had a beneficial effect on the product’s taste, color, fiber and protein content. Teterycz et al. [33] used hemp flour and hemp cake as an additive to semolina-based pasta, which allowed the pasta to become enriched with fiber (including insoluble dietary fiber (IDF) and neutral detergent fiber (NDF) fractions) and protein.

### 2.2. Fatty Acid Profile and Lipid Indices

Hemp oil is considered to be one of the most valuable edible oils because of the unique profile of its fatty acids (FA; Table 2) and their mutual relationships were analyzed concerning the lipid quality indicators, including the PUFA/SFA acid ratio, *n* − 6/*n* − 3 acid ratio, the atherogenicity index (AI), the thrombogenicity index (TI) and the hypocholesterolemic/hypercholesterolemic ratio (h/H).

**Figure 1:**
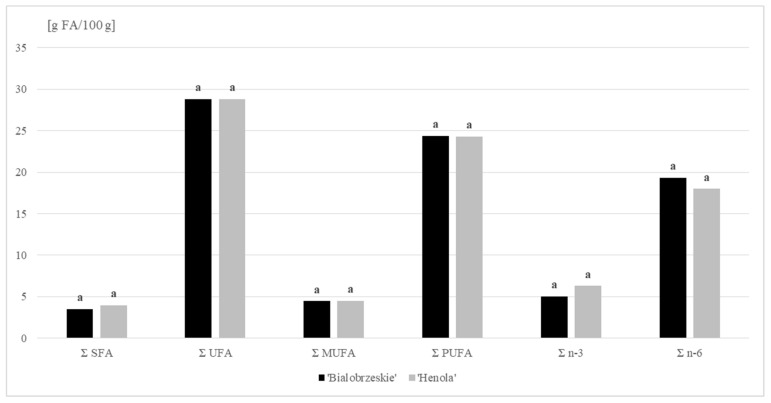
Fatty acids (FA) content (g/100 g DM) in ‘Bialobrzeskie’ and ‘Henola’ seeds.

Our research confirmed that hemp lipids are characterized by a high proportion of unsaturated acids (UFA). In the oil fraction extracted from seeds of the ‘Henola’ and ‘Bialobrzeskie’ varieties, the share of these acids accounted for, on average, 88 % of the total FA. Polyunsaturated fatty acids (PUFA) dominated the UFA profile. Their average percentage in total FA was as high as 75%, corresponding to a concentration of around 24 g PUFA/100 g (Figure 1). Linoleic acid (C18:2 *n* − 6 c; LA) belonging to this group accounted for 55% of the total FA content (mean value). The presence of α-linolenic acid (C18:3 *n* − 3; ALA) was also well emphasized among the PUFA acids, and was particularly evident in the lipid profile of the ‘Henola’ variety (ALA share >19%). When comparing the hemp varieties tested in terms of γ-linolenic acid (C18:3 *n* − 6; GLA), we found it to be twice as high in the lipid extract from the ‘Bialobrzeskie’ seeds (more than 3%). Interestingly, the FA identified in this variety also included four-unsaturated stearidonic acid (SDA), belonging to the *n* − 3 family. This 18-carbon PUFA is a direct product of the conversion of ALA catalyzed by Δ-6 desaturase. SDA is a component of many fish oils, but it is also found in plant seeds, especially the species of the Boraginaceae family. This compound is found in significant amounts in Ahiflower oil [34].

Oleic acid was the major MUFA acid in hemp seeds (mean value: 12%). The share of remaining compounds in MUFA, including *cis*-vaccenic, *cis*-11-eicosenoic and palmitoleic acids, did not exceed 1% of the total FA in any of the cases. In addition, trace amounts of erucic and nervonic acids were found in ‘Henola’ seeds (Table 2).

Saturated acids accounted for an average of 11% of the total FA, with only long-chain acids found in hemp seeds. C16:0 (palmitic acid) was their leading representative with a concentration averaging 2 g/100 g of the sample.

The lipid profile indices proved the nutritional value of hemp oil. Table 3 presents their values. The PUFA/SFA ratio and the *n* − 6/*n* − 3 ratio are used mainly to assess the effect of diet on the cardiovascular system. The PUFA ratio is based on the general assumption that PUFAs can reduce low-density lipoprotein (LDL) and serum cholesterol levels. In contrast, SFA has a hypercholesterolemic effect [35]. PUFA/SFA ratios exceeding 0.45 coupled with *n* − 6/*n* − 3 ratios below 5 are considered to be beneficial in the diet because of their high potential for preventing cardiovascular diseases. The PUFA/SFA ratio in the tested samples ranged from 6 to 7, whereas that of *n* − 6/*n* − 3 acids was approximately 3–4. Tringaniello et al. [36] found similar values of these ratios in hemp oils, indicating that a high proportion of polyunsaturated to saturated acids is considered beneficial for lowering serum cholesterol levels, reducing the risk of atherosclerosis and preventing heart disease. At the same time, the PUFA/SFA values determined in hemp oil in our study were much higher than those determined by Kim et al. [37] in olive oil (0.56), soybean oil (4.16), sesame oil (3.21), corn oil (4.25) or rapeseed oil (4.63).

The atherogenicity index (AI) is a lipid index that is often used in assessing the nutritional quality of plants, algae, fish, meat, dairy products and others. It indicates the relationship between the sum of pro-atherosclerotic SFA (C12:0, C14:0, C16:0) and anti-atherosclerotic UFA. The lowering of total cholesterol and LDL-C fraction in plasma is linked to the consumption of foods characterized by low AI. Our research showed that this ratio was below 0.1 in the fat extracted from hemp seeds (Table 3). GC analysis of the fatty acid profile showed that myristic and lauric acids were not detected in the tested samples. The share of palmitic acid was 6.75 (‘Bialobrzeskie’) and 7.76% (‘Henola’), respectively. The total percentage of UFA exceeded these values by more than 10 times, which ultimately resulted in a very low AI for hemp oil. The determined thrombogenicity index (TI) also proved the high nutritional quality of the tested samples. The IT values indicated a clot formation tendency in blood vessels, taking into account the relationship between prothrombogenic fatty acids (C12:0, C14:0 and C16:0) and the anticoagulant MUFA and PUFA *n* − 3 and *n* − 6 family [38]. In both ‘Bialobrzeskie’ and ‘Henola’ hemp seeds, the calculated TI was 0.03. However, for the hypocholesterolemic/hypercholesterolemic (h/H) ratio, the values were 12.98 and 11.21, respectively.

Similar AI values for hemp oil were obtained by other authors, including Razmaite et al. [29]. For the TI and h/H ratios, Ying et al. [39] found higher values than those determined in our study. This was probably due to differences in the proportion of SFA, MUFA and PUFA *n* − 6 and *n* − 3 acids in hemp oils. From the analyses conducted by these researchers, it follows that the proportion of the FA mentioned above was 8.6, 10.7, 53.2 and 25.4%, respectively [39]. Except for the proportion of PUFAs from the *n* − 3 family, these values are lower than our study found.

### 2.3. Amino Acids Profile and Content

Table 4 summarizes the determined contents of 17 amino acids concerning the hemp seed weight (g/100 g DM of seeds) and hemp protein (g/100 g protein). In terms of the contents of essential amino acids, the raw materials studied were compared to a theoretical reference protein for people >1 year of age, according to the Institute of Medicine (IOM; Washington, DC, USA), in addition to egg protein (FAO/WHO) [40].

Protein quality is determined by the content of essential, exogenous amino acids. Based on this criterion, proteins from products of animal origin, such as eggs, milk, dairy products and meat (including poultry and fish), are treated as a complete protein. On the contrary, most plant proteins, due to their lower contents of essential amino acids—lysine, tryptophan, methionine and valine—are classified as incomplete. The amino acid composition of hemp protein draws our attention because of the perfect match of its Thr and Leu contents with those of the reference protein. According to IOM experts, the reference (theoretical) protein with a composition of essential amino acids that is optimal for humans contains 2.7 g Thr and 5.5 g Leu/100 g of protein (Table 4), which is precisely the amount calculated for the protein of the ‘Henola’ variety seeds. For the ‘Bialobrzeskie’ variety, the Thr content was slightly higher at 3.05 g/100 g. As shown in Table 5, the limiting amino acid in the hemp protein is Lys. In terms of the Tyr + Phe (average content for both varieties 6.21 g/100 g) and His (2.33 g/100 g) contents, hemp protein exhibited a great degree of similarity to egg protein, which is valued for its high biological value.

Similar to many commercially available protein-rich plant products, such as broad beans, lupins, flax and rapeseed, hemp seeds are characterized by high contents of Glu, Arg and Asp. Due to the acid hydrolysis, glutamine and asparagine were completely converted into glutamic acid and aspartic acid. This also explains the high contents of amino acids that were found. The analyzed varieties of *C. sativa* contained, on average, 14.91, 10.10 and 8.91 g of these essential amino acids, respectively, per 100 g of protein. These values are similar to those obtained by Wang et al. [28]. Interestingly, these authors studied hemp protein isolates (HPI) and compared their amino acid compositions with FAO/WHO requirements for children aged 2–5 years. They found that, except for lysine and sulphur amino acids, the studied HPIs met the requirements for the age group mentioned above. Moreover, given its very high digestibility (88–91%) and its amino acid profile, hemp protein isolates can successfully compete with soy isolates [28]. As a result, these preparations have application potential in the food industry, especially plant-based substitutes for meat and dairy products.

## 3. Materials and Methods

### 3.1. Reagents and Analytical Standards

FMOC (9-fluorenylmethyl chloroformate in acetonitrile) and OPA (ortho-phthaldialdehyde and 3-mercaptopropionic acid in borate buffer) from Agilent Technologies (Santa Clara, CA, USA) were used as derivatization reagents in the analysis of amino acids. An amino acid kit was purchased from Agilent Technologies (Waldbronn, Germany).

For fatty acids’ determination, Supelco 37 Component FAME Mix (Supelco, Bellefonte, PA, USA) was used as a certified reference material (TraceCERT^®^). Boron trifluoride-methanol solution (~10%, for GC derivatization), methanol and water (suitable for HPLC) were purchased from Sigma-Aldrich (Merck, Darmstadt, Germany). n-Hexane 99% (suitable for Liquid Chromatography and UV-Spectrophotometry) was obtained from Macron Fine Chemicals^TM^ (Avantor, Center Valley, PA, USA).

### 3.2. Plant Material

The test material consisted of unshelled seeds of industrial hemp (Cannabis sativa L. var. sativa) of the ‘Henola’ (‘Zolotonowska 13’ × ‘Zenica’) and ‘Bialobrzeskie’ ((‘LKCSD’ × ‘Kompolti’) × ‘Fibrimon’) varieties purchased from the Institute of Natural Fibers and Medicinal Plant (INFMP, Poznań, Poland)/Polish Hemp Program in June 2020. After harvesting, the hemp seeds were dried with air (30–35 °C) to the humidity of approx. 8–11% and stored in a dry and airy room with humidity <10%.

The tested varieties were grown at the Institute of Natural Fibers and Medicinal Plants in Poznań. According to the specification given by INFMP, they are monoecious hemp with a high and stable degree of monoicy. They are central-European forms. Their growing season is adapted to Polish soil and climate conditions. For the ‘Bialobrzeskie’ variety, the growing period is 142–145 days after sowing (DAS). The flowering period falls into the 70–75 DAS period. Ripe crops reach 250–350 cm in height, and their seed yield ranges from 0.8 to 1.0 t/ha. The growing period of the ‘Henola’ variety is estimated at 115–120 DAS and the flowering period at 48–55 DAS. The plant height is 170–200 cm and the seed yield is 1.5–2.1 t/ha. In March 2021, both varieties were officially approved for cultivation also in the territory of Canada [41].

### 3.3. Labelling Nutrition and Energy Value of Seeds

Tests of the nutritional and energy value were carried out concerning the requirements of Regulation (EU) No 1169/2011 [42]. The hemp seeds were tested for energy value, protein content (N × 6.25; KjelFlex K-360, BÜCHI Labortechnik AG, Flawil, Switzerland), total fat (Soxhlet procedure; Automatic Soxhlet Extractor SOX606; Hannon Instruments, Jinan, China), digestible carbohydrates, total sugars, dry matter, salt and ash in accordance with AOAC methods (2007) [43]. The fiber content [44] was analyzed using the Bioquant 1.12979.0001 total dietary fiber enzymatic-gravimetric assay kit (Merck, Darmstadt, Germany).

### 3.4. Determination of the Fatty Acid Profile Using GC-FID

For fat extraction, hemp seed (±5.00 g) was ground and then homogenized with a chloroform: methanol mixture (2:1; *v*/*v*) with 0.001% antioxidant (butylhydroxytoluene-BHT). The solvent was evaporated in a nitrogen stream. The resulting crude lipid extract was saponified with 0.5 M KOH in methanol. The next stage included transesterification of fatty acids with the BF_3_ (boron trifluoride) solution in methanol as per the official AOCs Ce 2–66 method [45].

The conditions for chromatographic separation conformed with the procedure described by Wołoszyn et al. [46]. The methyl esters of fatty acids were quantified by GC-FID (Agilent 7890 A series, Agilent Tech. Inc.) using a J&W Scientific HP-88 series 100 m × 0.25 mm × 0.20 μm fused silica capillary column (Agilent Tech. Inc., St. Clara, CA, USA) and a flame-ionization detector (FID) from Agilent Tech.

### 3.5. Calculation of Lipid Health Indicators

Based on the analysis results for the fatty acid profile, selected nutritional quality parameters of the fat extracted from hemp seed were calculated. The following formulas were used for the calculations:

(1) PUFA/SFA ratio = (ΣDiUFA + ΣTriUFA + ΣTetraUFA)/ΣSFA [35];
(2) *n* − 6/*n* − 3 PUFA ratio = (C18:2 *n* − 6 + C18:3 *n* − 6)/(C18:3 *n* − 3 + C18:4 *n* − 3) [35];
(3) Atherogenicity Index AI = (C12:0 + 4 × C14:0 + C16:0)/Σ UFA [35];
(4) Thrombogenicity Index TI = (C14:0 + C16:0 + C18:0)/[(0.5 × MUFA) + (0.5 × Σ *n* − 6) + (3 × Σ *n* − 3) + (Σ *n* − 3/Σ *n* − 6)] [38];
(5) Hypocholesterolemic/Hypercholesterolemic Index h/H = [(C18:1 *n* − 9 + C18:1 *n* − 7 + C18:2 *n* − 6 + C18:3 *n* − 6 + C18:3 *n* − 3 + C20:3 *n* − 6 + C20:4 *n* − 6 + C20:5 *n* − 3 + C22:4 *n* − 6 + C22:5 *n* − 3 + C22:6 *n* − 3)/(C14:0 + C16:0)] [47]

### 3.6. Determination of the Amino Acid Content with the HPLC-DAD

The content of amino acids in hemp seeds was determined using high-performance liquid chromatography (HPLC) with the Agilent 1100 Series equipment coupled with the UV DAD detector and the reverse-phase AAA Eclipse Zorbax column (Agilent Technology Inc., St. Clara, CA, USA), 3.0 × 150 mm, 3.5 μm protected with an Agilent Technology column. The sample preparation was described in detail in the analytical procedure of Commission Regulation (EC) No 152/2009 [46].

The conditions for chromatographic separation of amino acids conformed with the ones proposed by Haraf et al. [48].

Cysteine was expressed as cysteic acid and methionine as methionine sulphone.

### 3.7. Calculation of AAS (Amino Acid Score)

The AAS was calculated using the reference protein scoring pattern of persons >1 year of age (Institute of Medicine IOM, US) according to the following equation from Haraf et al. [48]:

AAS = [(g of amino acid in 100 g of a test protein/g of amino acid in 100 g of requirement pattern)] × 100%

The amino acid with the lowest percentage is called the limiting amino acid.

### 3.8. Statistical Analysis

Nutrition value and chromatographic data are presented as the mean ± SD (*n* = 3). Statistical analysis was performed using the Duncan test and one-way ANOVA. *p*-value < 0.05 was considered to indicate a statistically significant difference.

## 4. Conclusions

Hemp is undoubtedly one of the crop plants with the most promising and versatile possible applications. Its seeds constitute a rich source of, among others, valuable protein and PUFA fatty acids with an excellent profile and exhibit great application potential not only in the production of oil but also a wide range of plant-based alternatives to animal-origin foods. As it follows from our research, hemp of the ‘Bialobrzeskie’ and ‘Henola’ varieties is a noteworthy raw material for food industry stakeholders who want to design modern, hypoallergenic plant food with outstanding nutritional value. Not only is its protein highly concentrated in seeds (>23%), but it also has an interesting amino acid profile with many features in common with both the reference protein that is recognized by experts as well as egg protein. The fat content exceeding 30% with excellent values of the nutritional indices provides yet another unquestionable advantage of the studied varieties. However, the ‘Henola’ variety seems to be preferable for introduction and cultivation due to its shorter growing period and higher seed productivity These facts encourage further research on hemp seeds aimed at producing plant food and promoting this raw material.

## Figures and Tables

**Table 1:** Nutritional and energy value of hemp seeds.

Parameter		HEMP	
		‘Bialobrzeskie’	‘Henola’
Energy value	kJ/100 g	1860 ± 18.83 a	1905 ± 11.31 a
Energy value	kcal/100 g	451 ± 4.46 a	461 ± 2.74 a
Dry matter	%	91.38 ± 2.25 a	92.44 ± 0.64 a
Protein *	%	23.54 ± 0.59 a	23.39 ± 0.38 a
Total sugars *	%	2.01 ± 0.11 a	2.09 ± 0.01 a
Digestible carbohydrates *	%	2.02 ± 0.24 a	4.48 ± 0.25 b
Dietary fiber *	%	28.88 ± 0.18 a	27.42 ± 0.34 b
Fat (total) *	%	32.28 ± 0.41 a	32.75 ± 1.70 a
Saturated fatty acids (SFA) *	g/100 g	3.48 ± 0.40 a	3.98 ± 0.25 a
Ash (total) *	%	4.66 ± 0.47 a	4.40 ± 0.16 a

a, b—mean values within a row with different letters are significantly different at *p* < 0.05; * according to the dry matter (DM) of seeds.

**Table 2:** Fatty acid (FA) profile/content in hemp seeds.

Fatty Acid	Chemical Structure	‘Bialobrzeskie’	‘Henola’	‘Bialobrzeskie’	‘Henola’
		(%) in total FA		(g/100 g DM of Seeds)	
palmitic	C16:0	6.75 ± 0.71 a	7.76 ± 0.89 a	2.18	2.54
palmitoleic	C16:1 *n* − 7	0.16 ± 0.05 a	0.16 ± 0.02 a	0.05	0.05
margaric	C17:0	0.07 ± 0.00 a	0.06 ± 0.00 a	0.02	0.02
stearic	C18:0	2.65 ± 0.08 a	2.84 ± 0.11 a	0.86	0.93
oleic	C18:1 *n* − 9	12.28 ± 1.75 a	11.95 ± 1.37 a	3.96	3.91
*cis*-vaccenic	C18:1 *n* − 7 (11 *cis*)	0.96 ± 0.07 a	0.96 ± 0.09 a	0.31	0.31
linoleic	C18:2 *n* − 6	56.46 ± 2.40 a	53.35 ± 3.01 a	18.22	17.48
γ-linolenic	C18:3 *n* − 6	3.33 ± 0.41 a	1.56 ± 0.13 b	1.07	0.51
α-linolenic	C18:3 *n* − 3	14.60 ± 1.85 a	19.15 ± 2.06 a	4.71	6.27
stearidonic	C18:4 *n* − 3	0.87 ± 0.11 a	0.00 ± 0.00 b	0.28	0.00
arachidic	C20:0	0.77 ± 0.09 a	0.86 ± 0.11 a	0.25	0.28
*cis*-11-eicosenoic	C20:1	0.34 ± 0.06 a	0.43 ± 0.04 a	0.11	0.14
eicosadienoic	C20:2	0.06 ± 0.01 a	0.06 ± 0.00 a	0.02	0.02
behenic	C22:0	0.31 ± 0.05 a	0.34 ± 0.06 a	0.10	0.11
erucic	C22:1 *n* − 9	0.00 ± 0.00 b	0.07 ± 0.00 a	0.00	0.02
lignoceric	C24:0	0.14 ± 0.00 a	0.17 ± 0.00 b	0.05	0.06
nervonic	C24:1 *n* − 9	0.00 ± 0.00 b	0.07 ± 0.01 a	0.00	0.02

a, b—mean values within a row with different letters are significantly different at *p* < 0.05.

**Table 3:** Lipid health indicators of hemp seeds.

Quality Parameters	‘Bialobrzeskie’	‘Henola’
Σ *n* − 6/Σ *n* − 3	3.86 ± 0.33 a	2.87 ± 0.61 a
Σ PUFA/Σ SFA	7.06 ± 1.03 a	6.17 ± 0.73 a
AI	0.08 ± 0.01 a	0.09 ± 0.01 a
TI	0.03 ± 0.00 a	0.03 ± 0.00 a
h/H	12.98 ± 3.00 a	11.21 ± 1.82 a

a—mean values within a row are not significantly different (*p* < 0.05).

**Table 4:** Amino acid profile of hemp seeds.

Amino Acids	‘Bialobrzeskie’		‘Henola’		Protein Pattern IOM **	Egg protein
	Content in Seeds (g/100 g DM)	Content in Protein (g/100 g)	Content in Seeds (g/100 g DM)	Content in Protein (g/100 g)	Content in Protein (g/100 g)	
Lys *	0.754 ± 0.098 a	3.20	0.752 ± 0.098 a	3.22	5.1	6.3
Met *	0.497 ± 0.065 a	2.11	0.463 ± 0.060 a	1.98		
Cys *	0.330 ± 0.060 a	1.40	0.320 ± 0.060 a	1.37		
Met + Cys *	0.827	3.51	0.783	3.35	2.5	5.6
Asp	2.160 ± 0.280 a	9.18	2.020 ± 0.260 a	8.64		
Thr *	0.718 ± 0.093 a	3.05	0.648 ± 0.084 a	2.77	2.7	4.7
Ser	0.990 ± 0.130 a	4.21	0.920 ± 0.120 a	3.93		
Glu	3.590 ± 0.390 a	15.25	3.410 ± 0.380 a	14.58		
Pro	0.820 ± 0.110 a	3.48	0.810 ± 0.110 a	3.46		
Gly	0.930 ± 0.120 a	3.95	0.880 ± 0.110 a	3.76		
Ala	0.900 ± 0.120 a	3.82	0.860 ± 0.110 a	3.68		
Val *	0.970 ± 0.130 a	4.12	0.980 ± 0.130 a	4.19	3.2	6.9
Ile *	0.830 ± 0.110 a	3.53	0.800 ± 0.100 a	3.42	2.5	5.9
Leu *	1.310 ± 0.170 a	5.56	1.290 ± 0.170 a	5.52	5.5	8.5
Tyr *	0.571 ± 0.074 a	2.43	0.523 ± 0.068 a	2.24		
Phe *	0.890 ± 0.120 a	3.78	0.930 ± 0.120 a	3.98		
Tyr + Phe *	1.461	6.21	1.453	6.21	4.7	7
His *	0.565 ± 0.073 a	2.40	0.527 ± 0.068 a	2.25	1.8	2.3
Arg Trp *	2.480 ± 0.320 a 0.437 ± 0.060 a	10.54 1.86	2.260 ± 0.290 a 0.322 ± 0.065 a	9.66 1.38	0.7	1.5

Lys—lysine; Met—methionine; Cys—cysteine; Asp—aspartic acid; Thr—threonine; Ser—serine; Glu—glutamic acid; Pro—proline; Gly—glycine; Ala—alanine; Val—valine; Ile—isoleucine; Leu—leucine; Tyr—tyrosine; Phe—phenylalanine; His—histidine; Arg—arginine; Trp—tryptophan; * essential amino acids; ** persons > 1 year of age; a—mean values within a row are not significantly different (*p* < 0.05).

**Table 5:** Amino Acid Score (AAS) (%) for hemp protein according to protein pattern (IOM).

Essential Amino Acid	‘Bialobrzeskie’	‘Henola’
Ile	141.20	136.80
Leu	101.09	100.36
Lys	62.75	63.14
Met + Cys	140.40	134.00
Phe + Tyr	132.13	132.13
Thr	112.76	102.59
Trp	265.71	197.14
Val	128.75	130.94
His	133.33	125.00

## Data Availability

All relevant data for the preparation of this manuscript are given in the text. Raw data used for preparation of this manuscript are available on request from the authors.
